# Autotransplantation and Orthodontic Treatment after Maxillary Central Incisor Region Trauma: A 13-Year Follow-Up Case Report Study

**DOI:** 10.1155/2018/2039714

**Published:** 2018-01-18

**Authors:** Farzad Piroozmand, Hossein Hessari, Mohsen Shirazi, Pegah Khazaei

**Affiliations:** ^1^Department of Orthodontics, School of Dentistry, Tehran University of Medical Sciences, International Campus, Tehran, Iran; ^2^Research Center for Caries Prevention, Dentistry Research Institute, Tehran University of Medical Sciences, Tehran, Iran; ^3^Department of Orthodontics, School of Dentistry, Tehran University of Medical Sciences, Tehran, Iran

## Abstract

The anterior maxilla is the most prone region to the trauma during childhood, and tooth loss sometimes happens due to trauma. Replacing the missing teeth has always been one of the dentists' challenges in children and adolescents, since their dentofacial growth is not complete. Autotransplantation of mandibular premolars with two-thirds or three-quarters of root formation provides the best prognosis for the tooth survival. This case report describes the management of a 10-year-old boy suffering a severe dental injury who received the autotransplantation of the premolars from mandible to restore the space caused by trauma in maxillary central incisor region and a 13-year follow-up of the autotransplantation.

## 1. Introduction

Severe dental injuries can lead to the permanent loss of teeth. The anterior maxilla is the most traumatized region and the most accident prone age of trauma is between 8 and 12 years old when the incisors are erupted, and the periodontal ligament has the minimal resistance to the external forces [1–3]. Space maintenance of avulsed tooth is necessary until the completion of dentofacial growth. One of the treatment options is replacing the teeth using implants. However, implants are not recommended in these patients because of their residual facial growth. The other treatment options include prosthetic replacement and orthodontic space closure [4]. On the other hand, if donor teeth are available, autotransplantation can be considered as a possible option. Autotransplantation involves moving the tooth from one site to another in the same individual [5]. In cases with bone loss, transplanted tooth can induce bone formation and reestablishment of alveolar process [6]. Successful tooth transplantation can improve mastication, speech, esthetics, dentofacial growth, and arch form [7]. Transplantation of mandibular premolars with root formation of two-thirds to three-quarters and wide open apex provides the best prognosis for the pulp survival and reduces the risk of resorption [8–11]. This case report describes the management of a 10-year-old boy suffering a severe dental injury who received the autotransplantation of the premolars from mandible to restore the space caused by trauma in maxillary central incisor region.

## 2. Case Report

**Figure 1:**
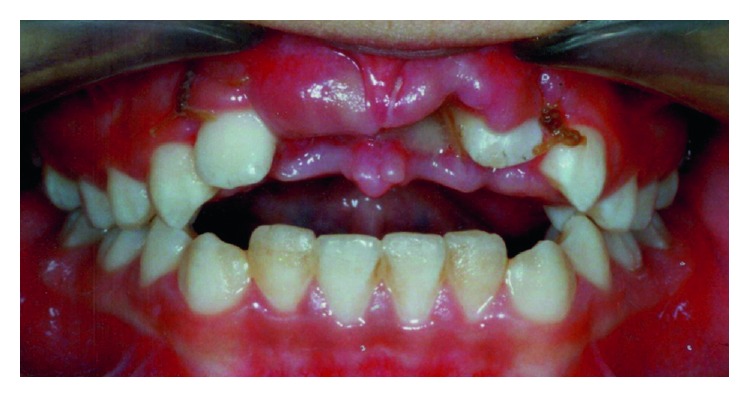
Clinical view showing the missing maxillary central incisors and intrusion of the maxillary lateral incisors.

A 10-year-old boy was brought to the Shariati hospital emergency department after a car accident. Avulsion of the maxillary central incisors and intrusion of lateral incisors were diagnosed (Figure 1). The avulsed central incisors were not replanted and missed.

**Figure 2:**
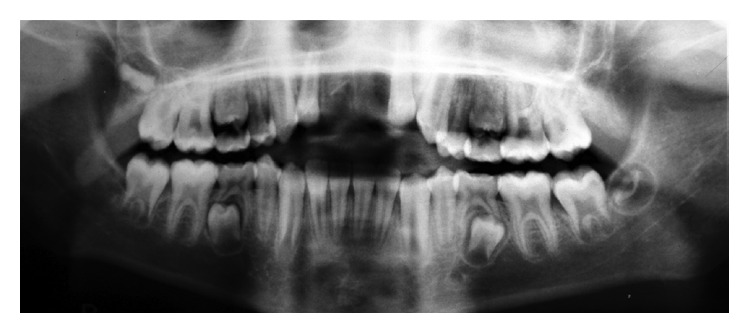
Panoramic radiograph before autotransplantation.

Panoramic radiograph showed a permanent dentition except second primary molars (Figure 2).

Four weeks after accident, the boy was examined by an orthodontist. Extraoral examination showed no soft tissue injury probably because of the time of examination. Intraoral examination also showed neither laceration nor alveolar bone fracture. Clinical examination revealed that the patient had an angle's Cl II malocclusion, open bite, and lower jaw space deficiency and crowding. The aim of orthodontic management was to treat the Cl II malocclusion and to relieve crowding by extractions in lower jaw. After the completion of radiographic and clinical examination, mandibular second premolars were selected for the extraction. Due to loss of maxillary incisors, we decided to transplant mandibular second premolars into the place of maxillary incisors at the time of orthodontics extractions.

## 3. Preliminary Orthodontics Treatment

**Figure 3:**
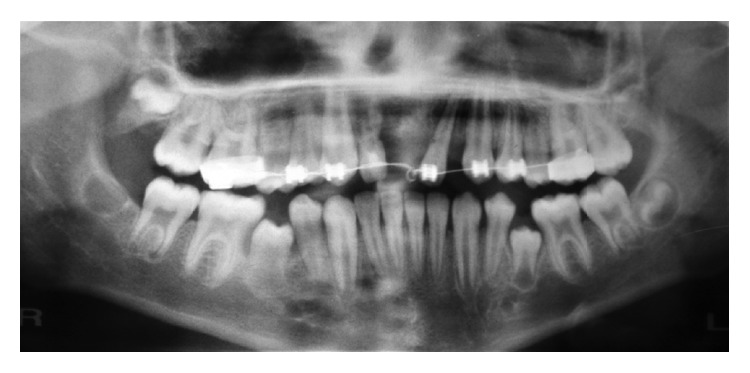
Radiographic view showing root resorption of maxillary lateral incisor.

Preliminary orthodontics treatment was performed to extrude the intruded maxillary lateral incisors and regain the incisal space. Evaluation of maxillary lateral incisors showed severe root resorption. Therefore, we had to extract them (Figure 3).

## 4. Teeth Preparation for Transplantation and Surgery

**Figure 4:**
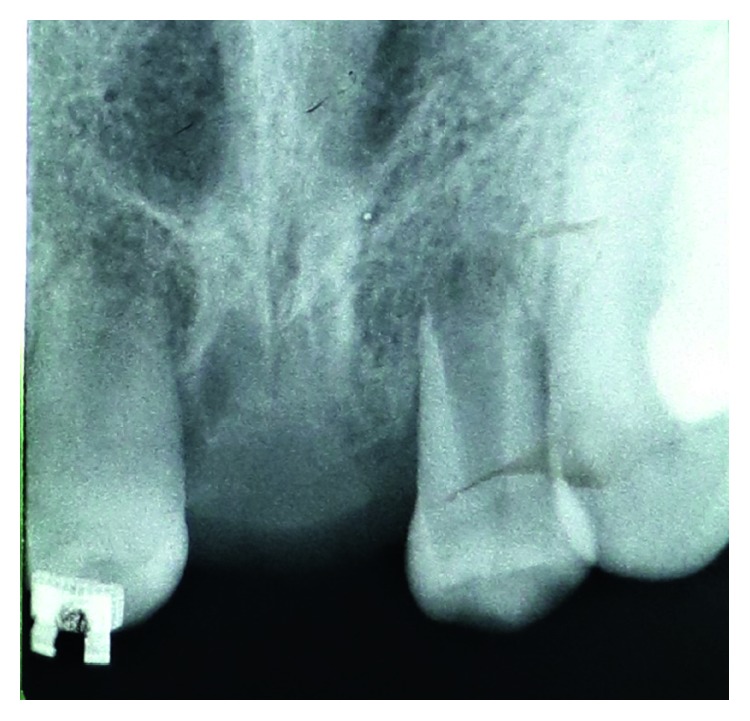
Transplanted mandibular second premolars.

The root formation of these premolars was two-thirds of its potential length at the extraction time. Prior to the extraction, with the aid of periapical radiograph, the size and length of premolar roots were measured, and then socket preparation was done in the maxillary central incisor region. A slightly larger socket was prepared, in order to minimize periodontal ligament damage, which could be happened because of excessive compressive forces or prolonged extra alveolar time for donor teeth. The premolars were extracted under the local anesthesia. A crevicular incision by number 12 scalpel blade was performed before the luxation to preserve the maximum possible periodontal ligament on the root surface. Teeth crowns were also covered by cotton rolls to avoid contacting the beaks of forceps with the root surface. After extraction, donor teeth immediately fitted into the prepared sockets (Figure 4). Proximal surface of premolars formed the labial surface of transplanted teeth in the incisor region.

## 5. Postsurgery Orthodontics Treatment

**Figure 5:**
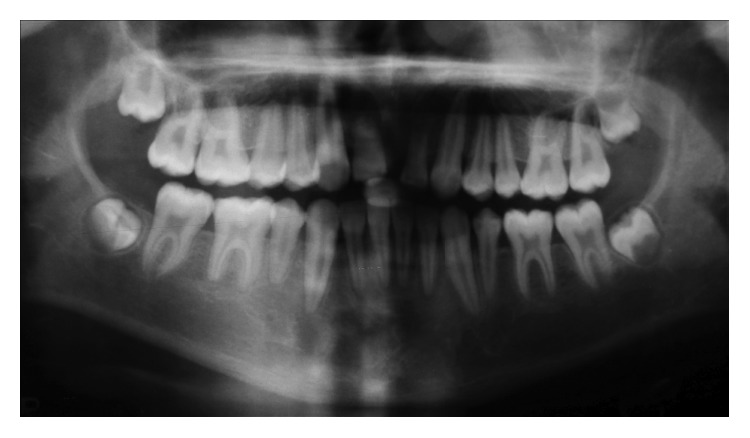
Radiographic view after orthodontic treatments.

**Figure 6:**
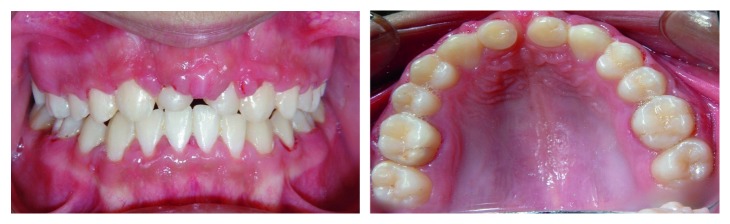
Clinical view after orthodontic treatments.

Transplanted teeth were splinted to the adjacent teeth using 0.3 mm thick stainless-steel wire and composite for 10 days. Five months after transplantation, transplanted teeth were tested clinically and radiographically. There was no sign of ankylosis, and root development had been initiated. Following that, orthodontics management started including fixing brackets on teeth and moving them as well as transplanted teeth. Root development of transplanted teeth continued, and they moved following orthodontic forces like other teeth. Periapical radiographs of transplanted teeth with 6 months' intervals revealed no sign of root resorption during treatment period. No inflammation of the gingiva or any patient complaint was reported. The treatment was accomplished to a Cl I occlusion with no prosthetic intervention during 23 months (Figures 5 and 6).

## 6. Follow-ups

**Figure 7:**
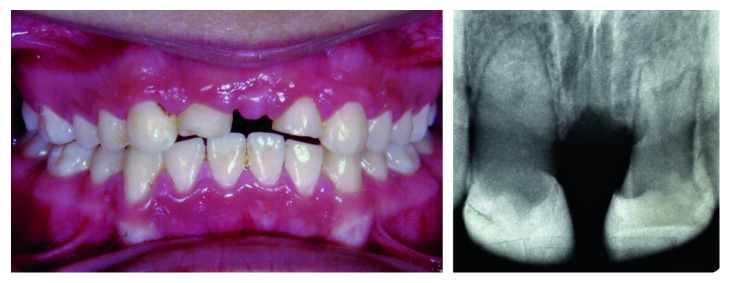
Radiographic and clinical view at the 6-year follow-up.

**Figure 8:**
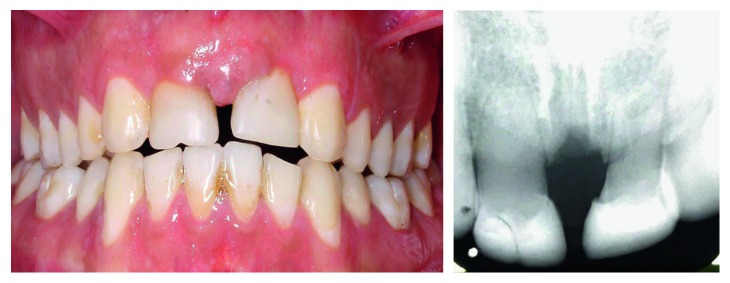
Radiographic and clinical view at the 13-year follow-up.

Six- and thirteen-year follow-ups confirmed complete health of transplanted teeth. The teeth were clinically vital, radiographically normal, with no pulp obliteration, and had no signs and symptoms of ankylosis or root resorption (Figures 7 and 8). Root development and the crown/root ratio were not similar in the transplanted teeth, although, periodontal ligament and pulpal health were acceptable in both. The alveolar ridge levels of transplanted teeth were parallel with that in adjacent teeth. However, there was a resorbed area in the midline due to large diastema between the transplanted teeth. No sign of mobilization was seen.

Further prosthodontic treatments or periodontal esthetic surgery could be performed at the later ages.

## 7. Discussion

Loss of maxillary incisors due to severe dental injuries is common, especially in ages between 8 and 12 years [1, 2]. Tooth loss during the growth time period can lead to the horizontal and vertical deficiencies of bone and soft tissue [10, 12].

There are several options to replace the missing tooth such as orthodontic treatment or implants. However, using implants is contraindicated until completion of the facial growth. When the implants are planned for the treatment, extensive surgeries like bone and soft tissue augmentation may be required [13].

Another possible treatment option is autotransplantation if the donor tooth is present. Transplanted tooth preserves alveolar bone until growth completion, even if future failure of transplantation occurred. Preservation of the bone will facilitate provision of implants. Also, the transplanted tooth can recover the proprioceptive function and normal periodontal healing. Therefore, a natural chewing feeling and natural biological response will occur. Additionally, the transplanted tooth can be used as a bridge abutment or orthodontic anchorage [14, 15].

The stage of root development is one of the main factors affecting the prognosis of the transplanted tooth [11, 16]. The chance of revascularization and reinnervation of the dental pulp is increasing when the apex is open. Transplantation of tooth with root development of more than 50% has shown high success rate [17]. Survival rate of transplanted premolars with incomplete roots is reported 95% to 98% up to 13 years by Andreasen et al. [18–20].

Ideally, when the donor tooth exhibits three-quarters development of full root length and diameter of apical opening is more than 1 mm at the time of autotransplantation, transplantation shows the most favorable prognosis [6, 16, 21]. In premolars, this developmental stage can be seen between ages of 10 and 13 years.

Regarding the transplantation stage, the results of Northway study [22] showed two-thirds and three-fourths completion of root development is the preferred stage for transplantation. The incidence of pulp necrosis and root resorption is greater when the apex of transplanted premolars is closed [10, 18, 19]. Transplantation of fully formed root decreases the potential for pulp regeneration, but an adequate endodontic therapy can ensure good prognosis of transplantation. In our case, transplantation was done when the root formation completed by three-quarters of its full length; and pulp healing and regeneration was achieved, as the root was immature. Transplanted tooth had a wide apical opening, and therefore, there was no need for endodontic treatment.

Another factor that affects the prognosis of transplantation is surgical procedure. A minimal handling and an atraumatic extraction to preserve the intact periodontal ligaments and Hertwig's root sheath should be considered. If not, ankylosis or root resorption and attachment loss might be occurred [10, 17, 21]. It is also important to minimize the implantation time after extraction. The donor tooth should be transplanted immediately after extraction to avoid drying out. However, Kim et al. showed no relationship between extraoral time and root resorption or ankylosis with a prolonged experimental time of 7.8 minutes [23]. In our case, the donor tooth was transplanted immediately after extraction, and maximum care was done to preserve the intact periodontal ligaments and Hertwig's root sheath.

Immobilization of the transplanted tooth is another factor that can influence the outcome of transplantation. However, long term and rigid immobilization can adversely affect the periodontal ligaments and pulpal healing of the transplanted tooth.

Orthodontic treatments can be initiated after confirmation of the presence of the lamina dura in radiographs and regeneration of periodontal space [24]. In our case, we started orthodontic treatment 5 months after transplantation. Orthodontic treatment ended 23 months after initiation. Pogrel [25] recommended that the final success or failure of transplanted tooth can usually be predicted at 2 years after transplantation. In our case, thirteen years after transplantation, no ankylosis or root resorption was observed.

## 8. Conclusion

In growing individuals, autotransplantation of immature premolar with open apex root can be considered as a predictable method to replace the missing teeth. Transplanted tooth can reestablish a normal alveolar process after loss of the bone due to dental injury.
